# TACE combined with sorafenib induces complete remission in an elderly patient with hepatocellular carcinoma, hepatic venous tumor thrombus, and diffuse lung metastasis: a case study and literature review

**DOI:** 10.3389/fonc.2025.1693092

**Published:** 2026-01-07

**Authors:** Zhao Guangsheng, Liu Song, Yin Shunhang, Ding Zhongyi, Tang Shunxiong, Zhou Jun, Zhang Yuewei

**Affiliations:** 1 Cancer Interventional Center, Affiliated Zhongshan Hospital of Dalian University, Dalian, Liaoning, China; 2 Dalian Medical University, Dalian, Liaoning, China; 3 Interventional Medicine Center, Linyi Cancer Hospital, Linyi, Shandong, China; 4 Hepatopancreatobiliary Center, Beijing Tsinghua Changgung Hospital, Beijing, China

**Keywords:** gelatin sponge particles, hepatocellular carcinoma, recombinant adenovirus p53 injection, sorafenib, transcatheter arterial chemoembolization

## Abstract

Hepatocellular carcinoma (HCC) with hepatic venous tumor thrombus (HVTT) and synchronous lung metastases has a poor prognosis and limited treatment options. We present a 70-year-old male with chronic hepatitis B and BCLC stage C HCC, featuring a large liver tumor (14×12×10 cm), HVTT, and diffuse lung metastases. The patient was treated with transarterial gene embolization (TAGE) using gelatin sponge microparticles and rAd-p53 (Gendicine^®^), followed by sorafenib. Imaging showed rapid tumor necrosis at 4 days, significant reduction of lung nodules at 4 weeks, and complete tumor necrosis with resolution of lung metastases at 15 weeks. The patient achieved complete remission and remains alive with 14-year survival.

## Introduction

Hepatocellular carcinoma (HCC) ranks among the most prevalent and life-threatening malignancies globally, with approximately 780,000 annual deaths attributed to liver cancer, half of which occur in China (1). Despite advances in management, the 5-year survival rate for HCC remains at approximately 12.5% (2). In China, over 80% of HCC patients are diagnosed at a stage where surgical resection is no longer feasible; thus, non-surgical treatments such as transcatheter arterial chemoembolization (TACE) have emerged as the core therapeutic modalities for this disease. The therapeutic value and positioning of TACE across different disease stages are explicitly outlined in various clinical practice guidelines (3–5). The incidence of HCC combined with HVTT is only 5.4%, but the incidence of lung metastasis is as high as 31.3% (6). HCC with HVTT or lung metastases still remains a challenge in management. In this report, we describe a rare case of elderly HCC, Imaging suggested that HCC not only invaded the hepatic veins, multiple diffuse miliary metastatic lesions in both lungs, What makes us very excited is that complete disappearance of the foci after the TAGE combined sorafenib, and the patient has a long-term survival.

## Patient information

A 70-year-old male patient with a diagnosis of advanced hepatocellular carcinoma (HCC) and a history of chronic hepatitis B virus (HBV) infection presented to the Interventional Radiology Department of our hospital for treatment.

## Clinical findings

In 2011, a CT scan during the patient’s physical examination revealed a large space-occupying lesion in the liver, which was considered a malignant lesion. At this time, the patient presented with significant abdominal distension and pain, and his dietary intake was also affected to some extent.

## Diagnostic assessment

**Figure 1:**
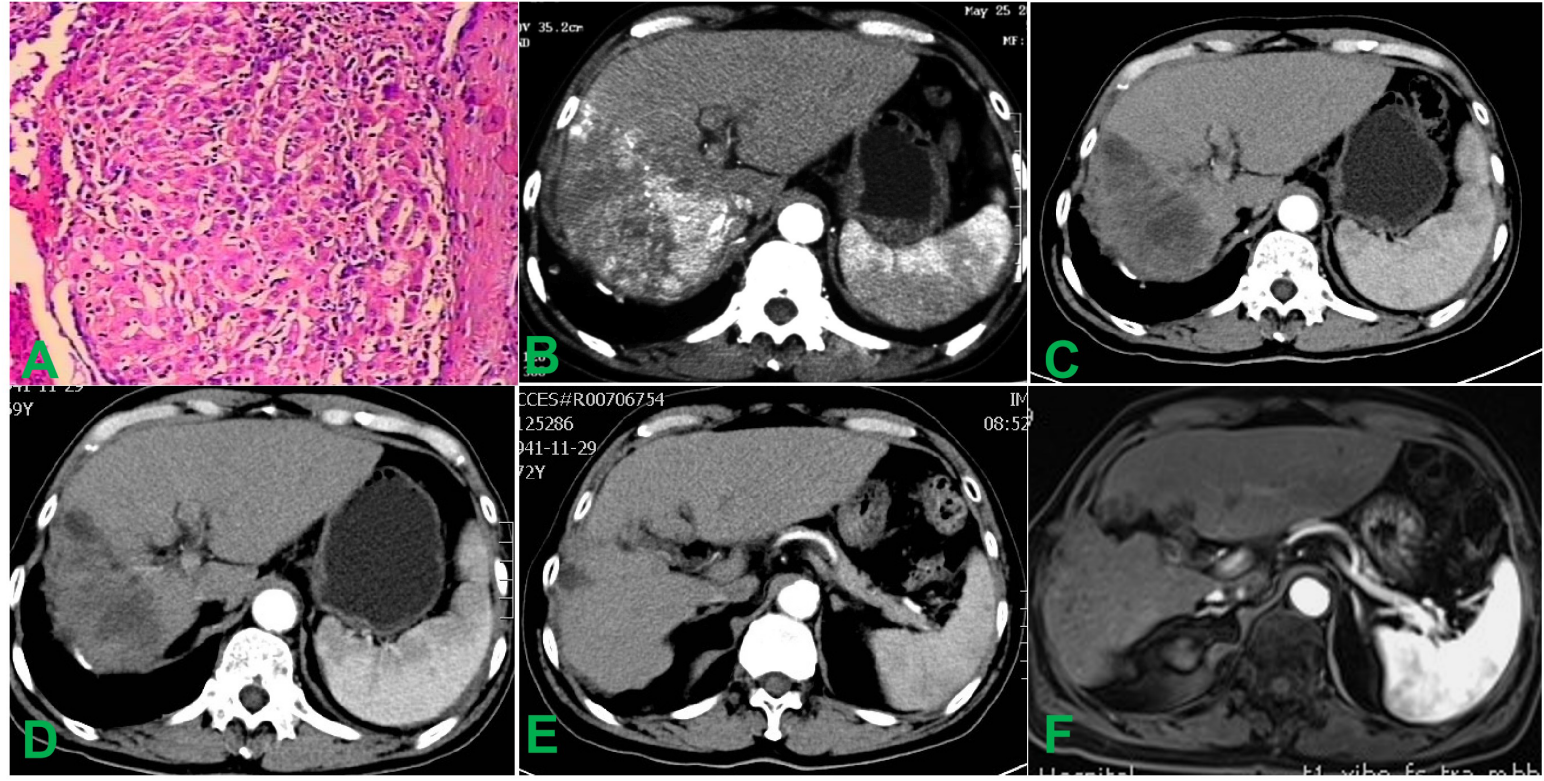
Histopathological and abdominal imaging findings before and after treatment. **(A)** Histopathological analysis of the biopsy specimen confirmed the diagnosis of hepatocellular carcinoma. **(B)** Pre-interventional contrast-enhanced CT scan demonstrates a 14.0 × 12.0 cm mass in the right hepatic lobe accompanied by tumor thrombus within the right hepatic vein. **(C)** Contrast-enhanced CT obtained 4 weeks post-intervention shows marked hypodensity throughout the lesion with the tumor size reduced to 10.0 × 6.0 cm, suggesting near-complete loss of enhancement. **(D)** At the 12-week follow-up, contrast-enhanced CT demonstrated the absence of significant enhancement in the hepatic tumor, consistent with complete necrosis, and showed a reduction in tumor size to 9.3 × 5.7 cm. **(E)** Surveillance imaging at 3 years post-intervention demonstrates near-complete resolution of the hepatic tumor and thrombus, with only minimal non-enhancing residual tissue. **(F)** Contrast-enhanced MRI performed 10 years after intervention confirms sustained intrahepatic stability without new or recurrent lesions.

**Figure 2:**
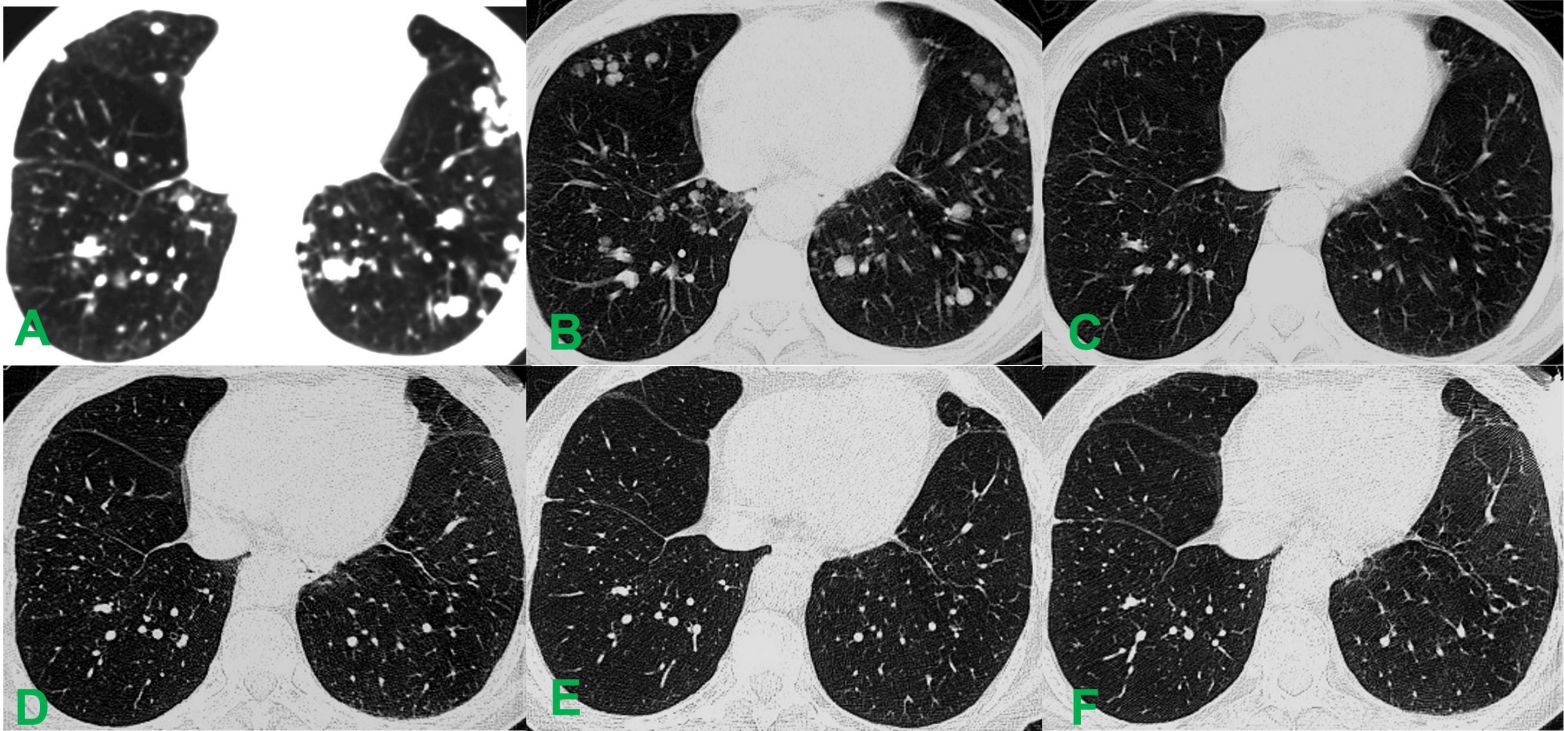
Comparative chest CT imaging before and after the intervention. **(A)** Pre-interventional pulmonary CT revealed numerous (>20) diffuse metastatic lesions in both lungs, with the largest measuring approximately 1.5 cm in diameter. **(B)** 4 weeks post-intervention, a reduction in both the number and volume of pulmonary metastatic lesions was observed, with the largest nodule measuring approximately 1.0 cm in diameter. **(C)** By 12 weeks after the procedure, the diffuse metastatic nodules had completely resolved. **(D)** No new lesions were detected on unenhanced CT at the 3-year follow-up. **(E)** Pulmonary CT imaging obtained 5 years post-intervention showed maintained remission. **(F)** At 10 years after treatment, follow-up CT demonstrated continued radiographic stability with no evidence of new or recurrent lesions.

Imaging studies indicated that the tumor was located in segments 7 and 8 of the liver, measuring 14×12×10 cm. The alpha-fetoprotein (AFP) level was 2107 ng/ml, with the formation of a hepatic venous tumor thrombus (HVTT) in the right branch. There were multiple diffuse nodules in both lungs, and the BCLC stage was C. Pathological biopsy of the liver tumor confirmed hepatocellular carcinoma (**Figures 1A, B**, **2A**).

## Therapeutic intervention

**Figure 3:**
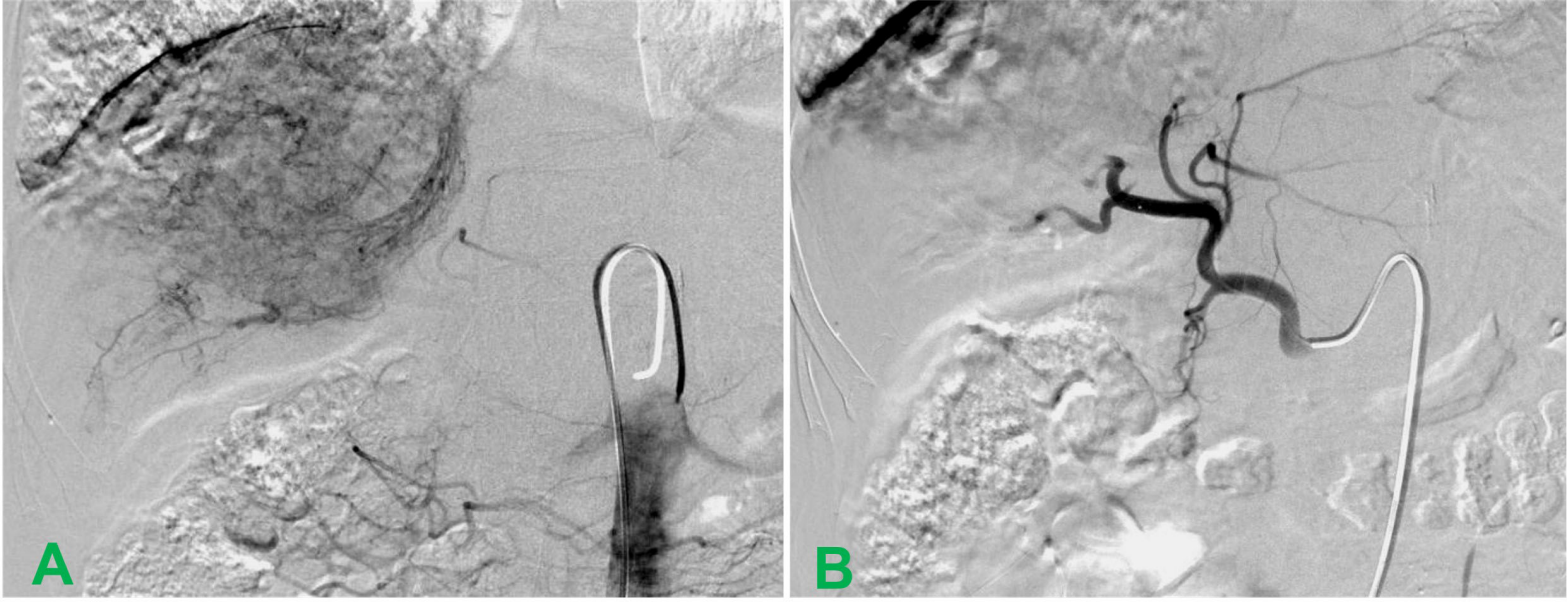
Hepatic arterial angiography performed during transarterial chemoembolization. **(A)** During the interventional therapy, the arteriography showed significant staining of the tumor and the hepatic vein carcinoma thrombus; **(B)** After interventional therapy, the branch of the right hepatic artery was truncated and the tumor staining completely disappeared.

During TACE, Seldinger,s method was used to puncture the right femoral artery, and a 5F-RH catheter was introduced into the right hepatic duct for routine celiac artery and common hepatic angiography. The results showed that the right hepatic artery had thickened, increased blood vessels, abundant arterial stage tumor staining, and strip staining of middle and advanced hepatic venous carcinoma (**Figure 3A**). The catheter was superselected into the right hepatic artery, rAd-p53 (5×1012 VP) (First Science & technology Middle Road, Shenzhen, Guangdong 518057, P. R. China)was diluted in 30 ml of saline and then mixed with 200mg of 350–560 μm GSMs (Gelfoam; Hanzhou alc Ltd., China). The above mixture were slowly embolized through the tumor target artery under fluoroscopy. When the flow rate of the contrast agent stopped, another angiography was performed after pausing for 2 minutes, and the embolization was stopped when the tumor staining completely disappeared (**Figure 3B**). During the procedure, the patient had mild pain in the gastric area and the pain was completely relieved after giving desoxoxin 10mg i. v. Six hours after surgery, the patient developed fever for 10 days as high as 39.1°C. After symptomatic treatment, it was gradually relieved. Liver-preserving and supportive therapy were given and was discharged 10 days after surgery. Oral sorafenib mesylate (200 mg/tablet, Nexavar^®^; Bayer Pharmaceuticals, Germany) was initiated on the third day following TACE. The initial dose was set at 400 mg twice daily.

## Follow-up and outcomes

Following TACE, the patient was enrolled into a standardized follow-up protocol to monitor treatment response and manage adverse events. Comprehensive disease evaluations were performed at 4 to 6-week intervals for the first 6 months, then every 3 months until 2 years post-TAGE, and subsequently extended to every 6 to 12 months thereafter based on sustained disease stability. Each follow-up visit included: 1) Clinical assessment: evaluation of symptoms and sorafenib-related adverse events (graded according to CTCAE v4.0); 2) Laboratory tests: complete blood count, hepatic and renal function panels, and serum alpha-fetoprotein (AFP) quantification to monitor biochemical response and organ function; 3) Imaging surveillance: chest non-contrast computed tomography (CT) and contrast-enhanced CT or magnetic resonance imaging (MRI) of the upper abdomen. Tumor response was assessed radiologically according to the modified Response Evaluation Criteria in Solid Tumors (mRECIST).

Serial changes in serum AFP, albumin (ALB), alanine aminotransferase (ALT), aspartate aminotransferase (AST), and total bilirubin (TBIL) levels at 4 and 7 days after treatment are summarized in **Table 1**. Contrast-enhanced CT at 4 weeks showed diffuse hypodensity throughout the lesion, accompanied by a mild reduction in the size of multiple miliary pulmonary metastatic nodules (**Figures 1C**, **2B**). By week 12, the hepatic tumor exhibited complete necrosis with an approximate 80% reduction in volume, and pulmonary metastases had nearly completely resolved (**Figures 1D**, **2C**). Subsequent follow-up imaging confirmed the disappearance of diffuse pulmonary metastases and progressive regression of intrahepatic lesions and tumor thrombi, culminating in complete remission.

**Table 1:** Changes in AFP and liver function indicators before and after treatment.

Time point	AFP(ng/ml)	ALT(U/L)	AST(U/L)	ALB(g/L)	TBIL(umol/L)
Preoperative	2107	68	55	39.3	21.6
4 days postoperative	956.7	294	469	32.6	35.4
7 days postoperative	562.1	121	99	34.4	30.5
4 weeks postoperative	135.4	48	80	43.6	17.4
7 weeks postoperative	32.11	32	53	46.0	23.5
11 weeks postoperative	4.86	16	42	40.6	18.4
15 weeks postoperative	1.45	18	44	43.4	16.5
3 years postoperative	3.21	23	38	39.6	11.5
5 years postoperative	2.95	20	40	43.2	17.4
10 years postoperative	1.01	19	30	44.0	10.6

**Figure 4:**
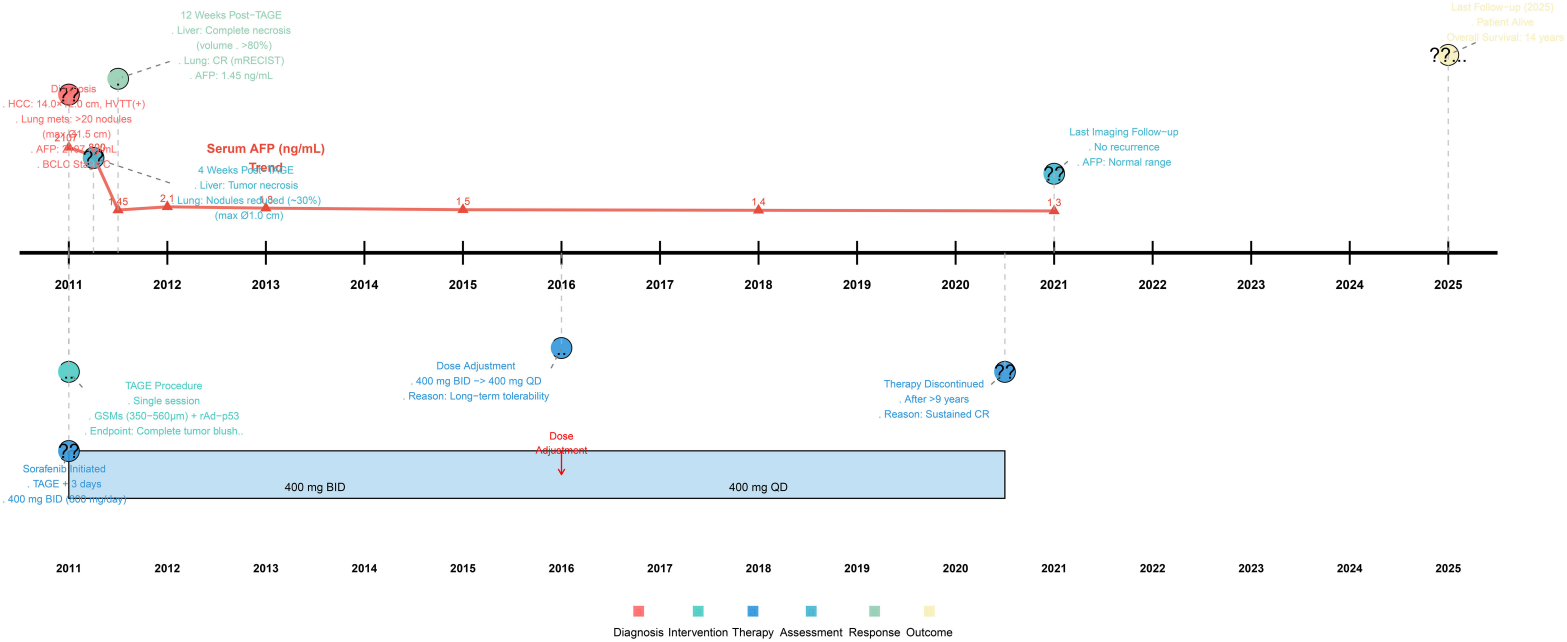
Timeline of clinical management and key outcomes.

Sorafenib dosing was managed proactively based on treatment response and tolerability. The initial dose of 400 mg twice daily (800 mg/day) was maintained until 2016. The most notable adverse event was a Grade 3 skin rash, which emerged early during treatment and persisted for approximately 8 weeks; it was managed with supportive care without requiring dose interruption. In 2016, after several years of sustained complete remission and considering the patient’s age and long-term tolerability, the dosage was reduced to 400 mg once daily (400 mg/day) following multidisciplinary discussion. This adjustment aimed to optimize long-term adherence and quality of life while preserving efficacy in a state of deep remission. Sorafenib therapy was ultimately discontinued in June 2020 after over 9 years of continuous administration, given the patient’s prolonged complete remission and excellent clinical condition. The patient remained alive at the last imaging follow-up in 2021. Subsequent regular telephone follow-ups (after the 2021 imaging assessment) confirmed that the patient continued to survive. Therefore, as of the latest follow-up in 2025, the patient has achieved an overall survival of 14 years since the initial diagnosis (**Figure 4**).

## Discussion

It is well-established that significant disparities exist between Eastern and Western treatment strategies for hepatocellular carcinoma (HCC) with portal vein tumor thrombus (PVTT) or lung metastasis (7, 8). In Europe and America, first-line treatment primarily consists of systemic therapies such as sorafenib or lenvatinib, which often serve as the sole therapeutic option for these patients. By contrast, in China, Japan, and Korea, surgical resection (SR) is a recommended modality for selected patients with resectable HCC accompanied by PVTT or lung metastases. Furthermore, with the progressive adoption of the Chinese treatment strategy, growing evidence from evidence-based studies has confirmed that the combination of transarterial chemoembolization (TACE) with systemic agents such as sorafenib yields superior outcomes compared to TACE or sorafenib alone (9, 10). The use of microparticle embolic agents mitigates the limitations associated with traditional embolic materials such as iodized oil (11), while recombinant adenovirus-p53 (rAd-p53) serves as an alternative to cisplatin, anthracycline, and other conventional chemotherapeutic agents (12, 13). This approach, referred to as transarterial gene embolization (TAGE), has demonstrated satisfactory clinical outcomes. The favorable short- and long-term efficacy observed in this case suggests that such results may not be an isolated clinical phenomenon, but rather indicative of an underlying reproducible mechanism. Several factors may account for this patient’s response.

First, effective control of the hepatic tumor constitutes the foundation of the treatment. In this case, the presence of HVTT not only defined an advanced anatomical stage but also signaled a highly invasive tumor phenotype, as HVTT is a recognized predictor of underlying microvascular invasion and satellite nodules, which are associated with a high risk of recurrence and metastasis (14). Therefore, an intensive local-regional approach was imperative. Both preoperative contrast-enhanced CT and interventional angiography revealed hypervascularity of the primary tumor and the thrombus itself. Complete occlusion of the tumor-feeding arteries and suppression of tumor neoangiogenesis thus represented critical prerequisites for reducing recurrence (15). In the TAGE procedure, 350–560 μm gelatin sponge microparticles (GSMs) were selected as the embolic agent. Their larger particle size compared to conventional iodized oil not only prevents pulmonary embolism via hepatovenous shunt but also enables comprehensive embolization of the tumor mass and its deep feeding arteries—including regional arteries supplying the liver tumor—thereby achieving maximal devascularization of the tumor (16). This profound devascularization is pivotal, as the hemodynamic status of HCC, reflected by parameters such as the portal venous and hepatic arterial coefficients, is intricately linked to long-term prognosis (17). The core mechanism of TACE lies in selectively occluding the tumor’s arterial supply. By achieving near-complete embolization of the hyper-arterialized tumor vasculature with GSMs, the TAGE procedure in this case may have fundamentally disrupted the aberrant hemodynamic microenvironment that fuels tumor growth and dissemination. This decisive alteration of the tumor’s blood supply—a key negative prognostic factor—likely laid a critical foundation for the subsequent systemic efficacy of sorafenib and contributed substantially to the patient’s long survival.

The achievement of complete tumor necrosis with a single TAGE session warrants specific analysis. Several intersecting factors likely contributed to this exceptional response. First, the tumor’s pronounced hypervascularity on angiography provided an optimal target for embolization, allowing the 350–560 μm GSMs to achieve deep and pervasive vascular occlusion. Second, the embolization endpoint was pursued rigorously until complete disappearance of tumor blush, ensuring maximal ischemic insult. Beyond these technical and anatomical considerations, unique aspects of the combined regimen and potential patient-specific factors may have played a role. The inclusion of the gene therapeutic agent rAd-p53, which induces p53-mediated apoptosis, could have synergized with ischemia to enhance tumor cell death. Furthermore, the subsequent systemic sorafenib therapy may have suppressed pro-angiogenic rebound and complemented local devascularization. Lastly, while the presence of hepatic vein tumor thrombus indicates an aggressive phenotype, the individual tumor biology in this case may have exhibited a heightened sensitivity to acute hypoxia/ischemia induced by embolization. Thus, the observed “single-session complete necrosis” likely represents the confluence of a radically effective embolization strategy, a multi-mechanistic combined treatment (gene therapy + targeted therapy), and a potentially favorable individual tumor response.

This observation was further corroborated by post-interventional imaging follow-up. Plain CT scan performed four days after TAGE revealed marked low-density alterations within the liver tumor and hepatic vein tumor thrombus, accompanied by a honeycomb-like necrotic pattern. GSMs are currently the only non-permanent embolic agent available. With a recanalization time of approximately two weeks, they not only ensure sustained necrosis of the liver tumor but also facilitate the restoration of blood flow to normal liver tissue. This provides a sound hepatic functional reserve for subsequent comprehensive treatment and avoids the exacerbation of cirrhosis associated with repeated TACE sessions using permanent embolic agents such as iodized oil (18).

Secondly, the administration of sorafenib played a pivotal role in preventing local tumor recurrence and treating diffuse pulmonary metastases. Recent research has further elucidated the synergistic mechanisms underlying such combination therapy. The concurrent use of targeted agents with TACE not only systemically inhibits the VEGF pathway but also enhances the durability of local treatment by modulating the tumor microenvironment (19). This mechanism aligns with the classic “post-embolization syndrome” theory: following complete embolization of the tumor-feeding arteries, levels of pro-angiogenic factors such as vascular endothelial growth factor (VEGF) are significantly elevated in peripheral blood and peritumoral tissues, contributing to an immunosuppressive microenvironment that promotes tumor recurrence and metastasis (20). As a multi-kinase inhibitor, sorafenib effectively suppresses tumor angiogenesis and cellular proliferation, thereby compensating for the limitations of TACE in treating large hepatocellular carcinomas. Evidence-based studies have confirmed that the combination of TACE and sorafenib synergistically controls disease progression in advanced HCC, improves prognosis, and prolongs survival—an effect described as “attenuated synergy” (21, 22). In the present case, the timely initiation of sorafenib following TAGE-induced profound devascularization likely acted through the dual mechanisms of “local adjuvant intensification” and “systemic control,” effectively counteracting the post-embolization surge in VEGF. This may have been instrumental in achieving complete regression of pulmonary metastases and establishing the foundation for long-term recurrence-free survival.

Earlier studies on TACE combined with sorafenib for HCC with lung metastasis reported an objective response rate of 20.0% and a cumulative disease control rate of 46.7% in pulmonary lesions; however, no complete remission (CR) was documented. In the present case, oral sorafenib was initiated three days after TAGE. After 15 weeks of continuous treatment, not only did the hepatic tumor and venous thrombus undergo complete necrosis, but multiple metastatic lesions in both lungs also gradually resolved completely. According to mRECIST criteria, CR was achieved for both the hepatic tumor and pulmonary metastases. Serum AFP levels declined from 2107 ng/mL to 1.45 ng/mL. The patient’s overall survival has reached 14 years. To our knowledge, such reports remain exceedingly rare.

Third, the incorporation of rAd-p53 gene therapy into TACE/TAE regimens warrants discussion. Although rAd-p53 has achieved only limited success in the treatment of solid tumors, its contribution to the exceptional short- and long-term outcomes in this patient cannot be ruled out. The necessity of chemotherapy in TACE remains controversial. Although regimens containing oxaliplatin and fluorouracil have been incorporated into guidelines for advanced HCC, considerable evidence suggests no significant difference in efficacy between TACE and bland TAE. This perspective, however, requires further validation through well-designed clinical trials. As a gene-correcting agent, rAd-p53 is capable of rectifying DNA damage and exerting antitumor effects at the genetic level. Unlike conventional chemotherapy, this process is not associated with systemic toxicity and may be regarded as a “green” antitumor strategy—though many mechanistic aspects remain unclear. Moreover, embolization with GSMs blocks arterial inflow to the tumor bed. When combined with a therapeutic agent such as rAd-p53, it may prolong local exposure and enhance adenovirus-mediated transduction of tumor cells, leading to higher exogenous p53 expression and potentially improved clinical outcomes (23).

Lastly, modulation of the immune microenvironment may have contributed to the enhanced efficacy and survival in this case. Tumor necrosis can release tumor-specific neoantigens, initiating a potent antitumor immune response. Studies indicate that TACE not only reduces tumor burden but also induces systemic antitumor immunity. It remodels immune cell populations in both peripheral blood and the tumor microenvironment, providing a rationale for combining TACE with immunotherapy. Our subsequent investigations revealed elevated levels of NK cells, NKT cells, CD4+ T cells, and an increased CD4+/CD8+ ratio, accompanied by decreased IL-17A and CD8+ T cells following TACE (24). Concurrent changes in immunoinflammatory factors within the tumor microenvironment (25) and a significant reduction in PD-1+ Treg cells, PD-1+ CD4+ T cells, and PD-1+ CD8+ T cells in peripheral blood after TACE have also been observed (26). These collective alterations in the immune microenvironment may potentiate antitumor immunity, offering an explanation for the remarkable phenomenon of complete regression of diffuse hepatic and pulmonary metastases following interventional therapy.

## Conclusions

The mechanisms underlying malignant tumors are highly complex. An overreliance on or overestimation of any single treatment modality is likely to lead to therapeutic dead ends. Therefore, a multimodal approach, combining various treatments that act through distinct mechanisms, remains the optimal strategy for managing advanced HCC. This integrated paradigm may represent an indispensable and prolonged phase in the evolution of HCC therapy. In the present case, the combination of TACE and sorafenib yielded highly satisfactory outcomes. Following only a single session of interventional therapy, not only were the primary liver tumor and associated venous tumor thrombus successfully mitigated, but the diffuse metastatic lesions in both lungs also gradually regressed and eventually disappeared completely. The patient achieved long-term survival with well-preserved quality of life. Owing to the complexity of the human immune network, the precise mechanisms of antitumor immunity remain to be fully elucidated. Nevertheless, this case offers valuable insights for the treatment of advanced HCC and brings hope that conquering this challenging malignancy may become an achievable goal in the future.

## Data Availability

The raw data supporting the conclusions of this article will be made available by the authors, without undue reservation.
